# Isotope labelling of Rubisco subunits provides *in vivo* information on subcellular biosynthesis and exchange of amino acids between compartments

**DOI:** 10.1111/j.1365-3040.2012.02485.x

**Published:** 2012-07

**Authors:** Doug K Allen, Russell W Laclair, John B Ohlrogge, Yair Shachar-Hill

**Affiliations:** 1 USDA-ARS, Plant Genetics Research UnitSt Louis, MO 63132, USA; 2 Donald Danforth Plant Science CenterSt Louis, MO 63132, USA; 3 Great Lakes Bioenergy Research CenterEast Lansing, MI 48824, USA; 4 Department of Plant Biology, Michigan State UniversityEast Lansing, MI 48824, USA

**Keywords:** compartmentation, isotopic labelling, metabolic flux analysis, primary metabolism

## Abstract

The architecture of plant metabolism includes substantial duplication of metabolite pools and enzyme catalyzed reactions in different subcellular compartments. This poses challenges for understanding the regulation of metabolism particularly in primary metabolism and amino acid biosynthesis. To explore the extent to which amino acids are made in single compartments and to gain insight into the metabolic precursors from which they derive, we used steady state ^13^C labelling and analysed labelling in protein amino acids from plastid and cytosol. Ribulose 1,5-bisphosphate carboxylase/oxygenase (Rubisco) is a major component of green tissues and its large and small subunits are synthesized from different pools of amino acids in the plastid and cytosol, respectively. Developing *Brassica napus* embryos were cultured in the presence of [U-^13^C]-sucrose, [U-^13^C]-glucose, [U-^13^C]-glutamine or [U-^13^C]-alanine to generate proteins. The large subunits (LSU) and small subunits (SSU) of Rubisco were isolated and the labelling in their constituent amino acids was analysed by gas chromatography-mass spectrometry. Amino acids including alanine, glycine and serine exhibited different ^13^C enrichment in the LSU and SSU, demonstrating that these pools have different metabolic origins and are not isotopically equilibrated between the plastid and cytosol on the time scale of cellular growth. Potential extensions of this novel approach to other macromolecules, organelles and cell types of eukaryotes are discussed.

## INTRODUCTION

Subcellular compartmentation is a defining characteristic of eukaryotic organisms. Organelles serve multiple purposes but foremost among these is the compartmentalization of metabolism. By segregating enzymes and metabolic fluxes into distinct compartments, more complex and sophisticated control over metabolism can be achieved (Winter, Robinson & Heldt 1993). In addition, anabolic and catabolic pathways can be separated to allow simultaneous synthesis and breakdown of metabolites. As one example, fatty acids can be synthesized in the plastids of plants while fatty acid degradation by beta-oxidation simultaneously takes place predominantly in the peroxisomes (Cooper & Beevers 1969; Germain *et al*. 2001; Baker *et al*. 2006; Khan, Adham & Zolman 2012) and possibly to a lesser extent in the mitochondria (Dieuaide *et al*. 1993; Masterson & Wood 2009). Separated anabolic and catabolic pathways also occur in multiple compartments for the metabolism of proteins (Vierstra 1993), purine bases (Zrenner *et al*. 2006) and carbohydrates (Zeeman *et al*. 2007a; Zeeman, Smith & Smith 2007b). The different organelle environments permit distinct metabolic fates for compounds based upon their location [e.g. compartmentation of glycolytic intermediates directed towards different products (Giege *et al*. 2003; Graham *et al*. 2007; Andriotis *et al*. 2010)].

A number of experimental and bioinformatic approaches are available to study the compartmentation of metabolism. Subcellular fractionation has been used to probe metabolism within individual compartments (Stocking 1959; Gerhardt & Heldt 1984; Winter *et al*. 1993). However, fractionation methods may alter metabolite concentrations and impact metabolic activity as small molecules can leak out of organelles during their isolation. Development of rapid isolation techniques with non-aqueous fractionation of organelles (Gerhardt & Heldt 1984; Gerhardt, Stitt & Heldt 1987; Stitt *et al*. 1989; Farre *et al*. 2001) has improved quantification of some of the more abundant and stable metabolites. Recent advances have included coupling this technology with metabolomics to provide new insights to metabolic function (Benkeblia, Shinano & Osaki 2007; Geigenberger, Tiessen & Meurer 2011). Nonetheless, the approach remains technically challenging and the extent to which metabolites may move between compartments during isolation remains uncertain despite the use of marker enzymes to identify organelles and determine the purity of separation.

Protein localization with fluorescent tags, immuno-tracking and sequence-based targeting predictions have provided information on subcellular localization of enzymes. However, many predictions are not unambiguous (see Supporting Information Table S1) and the presence of enzymes in a compartment determined from these methods does not quantitatively describe pathway activity. Overall, these methodological limitations make it challenging to evaluate many of the *in vivo* metabolic fluxes that occur within different subcellular compartments. This in turn limits our understanding of complex systems (Allen, Libourel & Shachar-Hill 2009a) and sometimes forces us to redefine descriptions of enzymatic roles (Schwender *et al*. 2004). These limitations impede the success of metabolic engineering strategies that require targeting of overexpressed enzyme activities to the appropriate subcellular location (Ma *et al*. 2000).

Many studies of metabolism utilize stable isotope labelling to infer fluxes. Incorporation of information based on subcellular synthesis of fatty acids, carbohydrates and other metabolites has resulted in models with increased sophistication for developing seeds of oilseed rape (Schwender, Ohlrogge & Shachar-Hill 2003; Schwender, Shachar-Hill & Ohlrogge 2006; Junker *et al*. 2007), sunflower (Alonso *et al*. 2007), maize (Alonso, Val & Shachar-Hill 2010, 2011), *Arabidopsis* seeds (Lonien & Schwender 2009), soybean (Sriram *et al*. 2004; Iyer *et al*. 2008; Allen, Ohlrogge & Shachar-Hill 2009b) and cells of *Arabidopsis* (Williams *et al*. 2008; Masakapalli *et al*. 2010). Information on compartmentation is also frequently deduced based on literature or sequence-based predictions, which can lead to errors, particularly in non-model species. Furthermore, direct labelling evidence is needed if compartmentalized fluxes are to be defined as literature information that establishes subcellular metabolic location does not establish values for fluxes but instead increases the number of parameters that must be modelled. Thus, the extent and accuracy of network structure and choice of the most informative labelled substrates are critical to model-derived conclusions about metabolism (Libourel & Shachar-Hill 2008; Masakapalli *et al*. 2010; Sweetlove *et al*. 2010).

In the past we have analysed and reported on several metabolites that have well-documented subcellular origins (Allen, Shachar-Hill & Ohlrogge 2007). For example, ^13^C isotopes of glucose that are incorporated into starch and cell wall monomers were used to assess the exchange of carbon in hexose phosphate pools between the plastid and cytosol (Allen *et al*. 2007); protein glycosylation provides another cytosolic reporter of hexose units (Sriram *et al*. 2007). Additionally, distinctive origins of cytosolic and plastid acetyl-CoA pools could be deduced from labelling of fatty acids because the biosynthesis up to 18 carbons in chain length takes place in the plastids of plants (Bao *et al*. 2000), whereas subsequent elongation to C20 and beyond occurs outside the plastid (Ohlrogge, Pollard & Stumpf 1978; Whitfield, Murphy & Hills 1993; Bao, Pollard & Ohlrogge 1998). Because acetyl-CoA is not transferred across membranes (Liedvogel & Stumpf 1982), different pools of acetyl-CoA for fatty acid biosynthesis were analysed through ^13^C labelling measurements (Schwender & Ohlrogge 2002; Allen *et al*. 2007).

Stable isotope labelling of proteins that are assembled from amino acid pools in different subcellular compartments is a previously untested source of compartmental information. For eukaryotes, most translation takes place in the cytosol using mRNA that originated in the cell nucleus. However, because plastids and mitochondria have maintained their own genomes throughout evolution, there are a number of proteins that are synthesized in these compartments (Unseld *et al*. 1997; Martin *et al*. 2002; Burger, Gray & Lang 2003). As examples, many photosynthetic proteins involved with light harvesting and ATP generation are produced in the chloroplast (Martin *et al*. 2002), while enzymes important to the electron transport chain such as cytochrome reductase/oxidase proteins and NADH dehydrogenases are assembled specifically in mitochondria (Wintz, Chen & Pillay 1989; Unseld *et al*. 1997). Biosynthesis of protein in a specific location will be based upon the amino acid pools in that location.

We hypothesized that examination of labelling of protein subunits synthesized in different locations might reveal variations in isotope enrichment of amino acids which in turn could provide insights into compartmentalized fluxes of subcellular metabolism. To assess this hypothesis, developing *Brassica napus* (rapeseed) embryos were labelled with [U-^13^C]-sucrose, [U-^13^C]-glucose, [U-^13^C]-glutamine or [U-^13^C]-alanine. Analysis of the large and small subunits of ribulose 1,5-bisphosphate carboxylase/oxygenase (Rubisco), translated in the plastid and cytosol, respectively, demonstrates that this approach can be informative about important intermediates of central metabolism as well as highlighting the simultaneous synthesis and turnover of amino acids in multiple compartments.

## RESULTS

A major objective of this study was to explore if novel information about compartmentation of plant metabolism can be obtained by comparison of proteins synthesized in the cytosol to those synthesized in the plastid. As an initial test of this approach, we focused on Rubisco, the most abundant soluble protein of plant leaves, and the enzyme responsible for a majority of all carbon fixation (Dhingra, Portis & Daniell 2004). The Rubisco structure, a 480 000 to 590 000 kDa complex, consists of eight large subunits (LSU) and eight small subunits (SSU), approximately 50–55 and 12–15 kDA in size, respectively. The LSU and SSU genes are encoded by the plastid and nuclear genomes, respectively (Blair & Ellis 1973; Gray & Kekwick 1974; Baker *et al*. 1975). The genes encoding SSU and LSU are transcribed from each genome and their mRNA is translated to protein by distinct ribosomal assemblies localized in each compartment. Therefore, the SSU protein is derived from amino acid pools present in the cytosol, whereas LSU protein synthesis depends on plastid pools of amino acids. After protein synthesis, the SSU is imported into the plastid where assembly of the complex takes place. Comparison of other pairs of proteins assembled in the cytosol versus the plastid might also reveal similar information. However, a major advantage of analysis of Rubisco is that because the SSU and LSU are components of a multi-subunit enzyme complex, with fixed stoichiometry, the timing of SSU and LSU protein synthesis is synchronized and temporal differences will be minimized.

**Figure 1:**
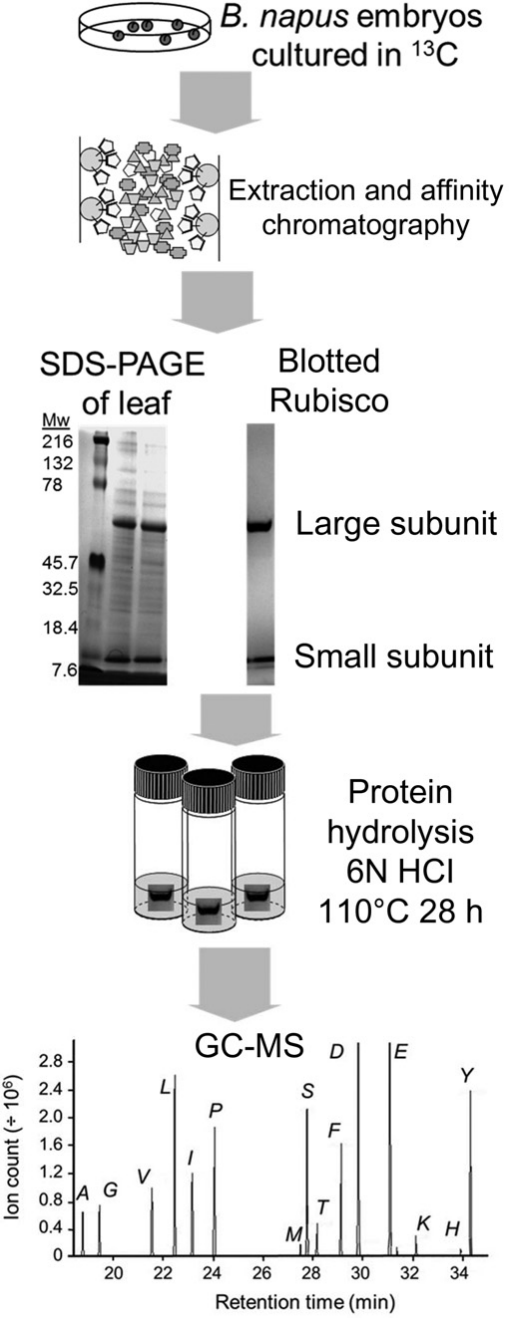
Schematic for analysis of ribulose 1,5-bisphosphate carboxylase/oxygenase (Rubisco) small and large subunits. *Brassica napus* embryos were cultured in ^13^C-labelled substrates, protein extracted and Rubisco was isolated by affinity chromatography. Sodium dodecyl sulphate–polyacrylamide gel electrophoresis (SDS-PAGE) was used to separate small and large subunits. Gels were electroblotted to polyvinylidene fluoride (PVDF) membrane, protein bands isolated and hydrolyzed with 6N HCl. Resulting amino acids were derivatized to the tert-butyl-dimethylsilyl products and analysed for isotopic enrichment by gas chromatography-mass spectrometry (GC-MS).

During development in siliques, *B. napus* embryos are green and express substantial levels of Rubisco (King, Badger & Furbank 1998; White *et al*. 2000; Ruuska *et al*. 2002; Ruuska, Schwender & Ohlrogge 2004). To analyse the SSU and LSU, we tested separation and isolation of subunits by gel electrophoresis with or without prior separation using molecular weight cut-off membranes. Although the subunits were well separated by polyacrylamide gel electrophoresis (PAGE), the recovery from crude extracts after electrophoresis resulted in LSU and SSU preparations contaminated with other proteins and in low yields. Therefore, we used antibody-coupled beads to isolate Rubisco from other proteins present in crude extracts (Fig. 1). LSU and SSU subunits of the affinity-purified Rubisco were then resolved by sodium dodecyl sulphate–polyacrylamide gel electrophoresis (SDS-PAGE). Direct protein hydrolysis of gel excised bands resulted in low amino acid yields and contaminants presumed to be amides that are a by-product of the polyacrylamide gel matrix. Therefore, protein was transferred to polyvinylidene fluoride (PVDF) membranes, hydrolyzed, and tert-butyl-dimethylsilyl derivatives (TBDMS) of amino acids were prepared and analysed by gas chromatography-mass spectrometry (GC-MS) (Fig. 1).

When developing *B. napus*, embryos are excised from siliques at an early stage of development and cultured with light (50 *µ*E); embryo development continues until mature viable seeds are produced with protein and lipid compositions similar to embryos that develop *in planta* (Schwender & Ohlrogge 2002). To mimic the *in planta* environment, media contained sucrose, glucose, glutamine and alanine at concentrations similar to those of the endosperm that surrounds embryos (Schwender & Ohlrogge 2002). The growth rate observed in this study was 0.3–0.4 mg DW day^−1^ which is comparable with previously reported growth in culture and *in planta* (Pomeroy *et al*. 1991; Schwender & Ohlrogge 2002). Media were supplemented with: [U-^13^C]-glucose, [U-^13^C]-sucrose, [U-^13^C]-glutamine or [U-^13^C]-alanine; substrates that enter metabolism through different reactions and transporters and are consumed at different rates. Therefore, each labelling experiment will result in unique metabolite enrichments and provide complementary information. Substrates were replaced with 100% [U-^13^C] label except the predominant carbon source for embryos, sucrose that was supplied as 1:1 combination of [U-^13^C] labelled and unlabelled sucrose. [U-^13^C] label was supplied for 2 weeks during which isotopic steady state conditions are observed (Schwender *et al*. 2003, 2006). Under these conditions, greater than 95% of total biomass accumulates during labelling, equivalent to 4.5–5 doublings. Therefore, the biomass analysed represents the steady-state metabolic labelling of metabolism during embryo development under conditions very similar to *in planta* growth.

Pools of metabolic intermediates may exchange between cytosol and plastid via the triose, hexose, pentose, four carbon and other transporters (Linka & Weber 2010). If exchange is rapid resulting in complete isotopic equilibration of these intermediates and/or amino acid pools between the cytosol and plastid, identical labelling patterns would be expected for proteins synthesized in these two locations. Here, equilibration refers to the labelling patterns in a particular amino acid becoming equal in plastid and cytosol through interchange of pools. The same result would be expected if synthesis of a metabolite only occurs in one compartment. When *B. napus* embryos were cultured with ^13^C labelled substrates, several amino acids of the large and small subunits of Rubisco were enriched differently. Of these experiments, [U-^13^C]-glucose and [U-^13^C]-sucrose provided the most statistically significant differences in labelling of LSU and SSU.

**Figure 2:**
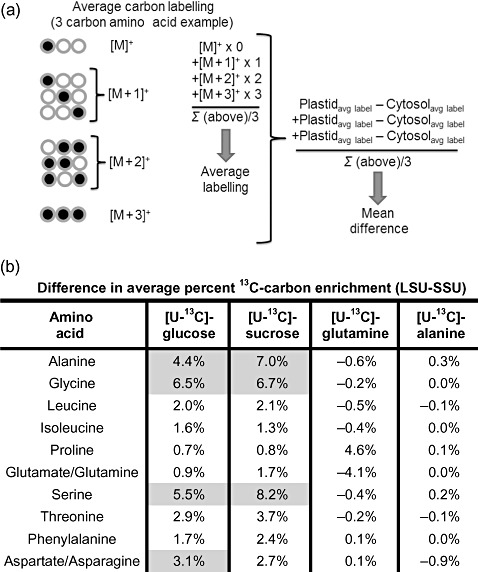
Differences in ^13^C enrichment of large subunit (LSU) and small subunit (SSU)-derived amino acids. Gas chromatography-mass spectrometry (GC-MS) results were subject to statistical comparison of the average carbon labelling for each amino acid. (a) The average labelling difference was calculated as shown by multiplying the fractional label of each mass weight by the number of labelled carbons for that mass weight, then summing these overall weights and dividing by the total number of carbons. The difference is defined as the average change in enrichment between the LSU and SSU fragments. (b) Differences in average carbon labelling of LSUs and SSUs for four labelling experiments. Significance was calculated using two-tailed Student's *t*-test with 99% levels shaded for minimum of *n* = 3 except for several amino acids within the alanine labelling experiment that were duplicates.

A comparison of the labelling in amino acids of LSU and SSU is presented in Fig. 2 and in the Supporting Information Tables S2–S6. Figure 2b presents the differences in label between LSU and SSU subunits for all experiments, with shading to indicate significance (*t*-test, two-tailed, 99%). Of the 20 amino acids present in protein, 10 could be analysed with high reproducibility and reliability. Asparagine and glutamine are deamidated during hydrolysis to their acidic counterparts; therefore ‘ASX’ and ‘GLX’ represent combined aspartate/asparagine and glutamate/glutamine. Cysteine and tryptophan are lost oxidatively during acid hydrolysis, and valine could not be resolved from an unidentified contaminant. Finally, methionine, lysine, arginine, histidine and tyrosine levels were too low to reliably analyse reflecting their lower abundance in protein and more challenging fragmentation patterns for GC-MS. The mass enrichments for different GC-MS fragments of the same amino acid are not independent, therefore the average labelling per carbon was used to compare LSU and SSU labelling (defined in figure caption). Additional average enrichment per carbon data is provided in Supporting Information Table S2.

### Alanine is simultaneously made in multiple compartments and is not isotopically equilibrated between the plastid and cytosol

**Figure 3:**
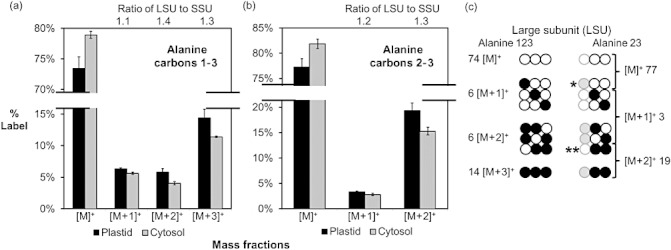
^13^C labelling differences in alanine of large subunits (LSUs) and small subunits (SSUs) of ribulose 1,5-bisphosphate carboxylase/oxygenase (Rubisco). Alanine enrichment varied between the cytosol and plastid pools as revealed by labelling with [U-^13^C]-glucose (*n* = 3, SD). Differences are reflected in isotopomers of both (a) three carbon and (b) two carbon gas chromatography-mass spectrometry (GC-MS) fragments. (c) Together these two fragments can be used to calculate the labelling of specific isotopomers that are indicated by (*) and (**). Alanine (LSU) labelled exclusively in the first carbon (*) represents 77 − 74 = 3% of all alanine isotopomers and is one half of the pool of singly labelled isotopomers. Enrichment of doubly labelled molecules in the second and third positions (**) is 19 − 14 = 5% out of a total pool of 6% and is the predominantly abundant isotopomer ([M+2]^+^ enrichment).

As indicated in Figs 2 and 3, after labelling with [U-^13^C]-glucose the enrichment of alanine was significantly different (*t*-test, 99%) in LSU (plastidic synthesis) compared with the SSU (cytosolic synthesis) (*n* = 3). Average ^13^C enrichment per carbon of alanine in the LSU was 21% and in the SSU was 16%, a relative difference of 22% and absolute difference of 4.4% (Fig. 2). Figure 3 presents two GC-MS fragments for alanine from [U-^13^C]-glucose labelling. Data derived from both the MS fragment representing all three of the backbone carbons of alanine and a second fragment (2nd and 3rd carbons of alanine) were consistent within each Rubisco subunit. The observed differences in labelling when [U-^13^C]-glucose was supplied were confirmed by the results of sucrose labelling experiments. These data indicate that alanine within the LSU is derived from precursors that are more highly labelled than alanine from the SSU protein. This provides a clear *in vivo* demonstration that cytosolic and plastidic pools of alanine are not in isotopic equilibrium.

As unlabelled alanine is provided in the media, this could highlight the disequilibrium by reducing the ^13^C enrichment of alanine in the cytosol compared with the plastid. Thus, a greater incorporation of unlabelled exogenous alanine into the cytosolic pool is the most straightforward explanation for the lower levels of SSU alanine ^13^C enrichment.

### Precursors for plastid and cytosolic alanine are distinct

Although the ^13^C distribution pattern in Fig. 3 was similar for LSU and SSU, statistically significant differences between the subunits were observed. If the differences between subunits (Fig. 2) were merely a result of dilution, then all labelled isotopomers for the SSU would be fractionally reduced to the same extent relative to the LSU. This is because the labelling profile of an unlabelled source of alanine will be composed of isotopomers exclusively of weight [M]^+^ (i.e. 100%) and 0% in [M+1]^+^ to [M+3]^+^ (after correction for natural abundance). Therefore, adding unlabelled alanine would increase the unlabelled [M]^+^ fraction and would uniformly decrease all other fractions. However, this was not observed. Instead, as indicated in Fig. 3, the ratio of LSU to SSU ^13^C enrichment varied in the different mass weights of alanine. The LSU to SSU enrichment for [M+1]^+^ was 1.1 whereas for [M+2]^+^ it was 1.4 (i.e. statistically significant fractional change of 30%), Fig. 3, and [M+3]^+^_LSU_/[M+3]^+^_SSU_ is 1.3. These differences reveal that relative fluxes through the alternative metabolic pathways are different in plastids and cytosol.

### Alanine labelling implies contributions of both glycolytic and pentose phosphate pathways

[U-^13^C]-hexose that is metabolized via glycolysis will retain equal ^13^C label in each carbon atom of the three carbon alanine backbone (reflected by the M+3 isotopomer of alanine). As indicated in Fig. 3, the [M+3]^+^ mass isomer represented only approximately 50% of the labelled [M+1]^+^, [M+2]^+^ and [M+3]^+^ mass isomers. The intermediate [M+1]^+^ and [M+2]^+^ mass isomers represent alanine molecules that contain both unlabelled and labelled carbons, which result from bond breaking of ^13^C backbone derived from the fully labelled hexose precursor. The analysis of fragment ions (Fig. 3) indicates that [M+2]^+^ isotopomers are predominantly labelled in the second and third positions of the alanine backbone. The breaking and reforming of bonds between carbons 1, 2, 3 and 4, 5, 6 of hexose occur in the pentose phosphate pathways (both oxidative and non-oxidative reactions) and through the Rubisco bypass (Schwender *et al*. 2004). The bypass incorporates carbon dioxide into 3-phosphoglyceric acid that can ultimately lead to alanine. These results imply substantial flux of hexose to pyruvate not only through glycolysis but also via the pentose phosphate or Rubisco bypass reactions. Thus, the enrichments are consistent with a significant Rubisco bypass contribution to phosphoglyceric acid production as previously observed (Schwender *et al*. 2004).

### *In vivo* labelling supports leucine biosynthesis in the plastid

Leucine is derived from pyruvate and acetyl-CoA. Proteomic [Supporting Information Table S1 (Zybailov *et al*. 2008)] and GFP studies (Maloney *et al*. 2010) indicate that committed enzymes in leucine biosynthesis are in the plastid (Binder 2010). Given the differences in alanine enrichment (that represents pyruvate), we examined whether leucine labelling demonstrated similar variation. Leucine enrichment in the LSU and SSU revealed no significant differences for any labelling experiment (Fig. 2). Thus, either the precursors of leucine (i.e. pyruvate and acetyl-CoA) are equivalently labelled across locations in metabolism, or leucine is made in one location and transported to other compartments.

**Figure 4:**
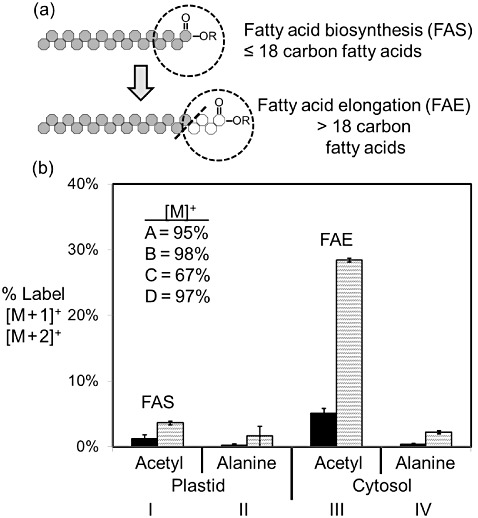
Labelling of fatty acid carbons from plastid and cytosolic reactions reflect distinct pools of acetyl-CoA. (a) The terminal acetate group of fatty acids synthesized in the plastid is enriched differently from fatty acids elongated in the cytosol (*n* = 3, SD). (b) In this experiment, embryo metabolism was inspected after labelling with [U-^13^C]-glutamine. The histogram presents the enrichment of four products of pyruvate including: acetyl-CoA from the plastid used in fatty acid biosynthesis (I), alanine from ribulose 1,5-bisphosphate carboxylase/oxygenase (Rubisco) large subunits (LSUs) (plastid synthesized) (II), acetyl-CoA from the cytosol used in fatty acid elongation (III) and alanine from Rubisco small subunits (SSU) (cytosol synthesized) (IV). The results indicate glutamine is not a significant source of carbon for fatty acid biosynthesis in the plastid (I) consistent with Schwender & Ohlrogge (2002) and Schwender *et al*. (2006) but provides carbon for fatty acid elongation steps (III). Glutamine is not a source of carbon for alanine biosynthesis in either location as indicated (II and IV).

To assess these two alternatives, we first analysed the labelling of plastidic and cytosolic acetyl-CoA using the McLafferty fragments of fatty acids as a reporter (Schwender & Ohlrogge 2002; Allen *et al*. 2007; Lonien & Schwender 2009). Synthesis of fatty acids up to 18 carbon chain lengths are derived from plastid acetyl-CoA whereas elongation of fatty acids for chain lengths greater than 18 carbons utilize acetyl-CoA from the cytosol (Ohlrogge *et al*. 1978; Whitfield *et al*. 1993; Bao *et al*. 1998; Schwender & Ohlrogge 2002). Figure 4 presents the ^13^C enrichment of terminal acetyl groups of C18 and C22 fatty acids after labelling with ^13^C-glutamine. The much higher enrichment (i.e. 31% average carbon label) in the C22-derived McLafferty fragment (i.e. the cytosolic acetyl group) than labelling of the C18 fatty acids (3%) reflects the compartmentation of the acetyl-CoA pools. Leucine was labelled to a similar extent as the C18 fatty acids, indicating that the plastid acetyl-CoA pool and not the cytosolic pool is the precursor of leucine. For reference, alanine from LSU and SSU is also presented. The low enrichments for both alanine pools indicate that the carbon skeleton of glutamine contributes little to alanine biosynthesis in agreement with previous work (Schwender *et al*. 2006; Allen *et al*. 2009b).

**Figure 5:**
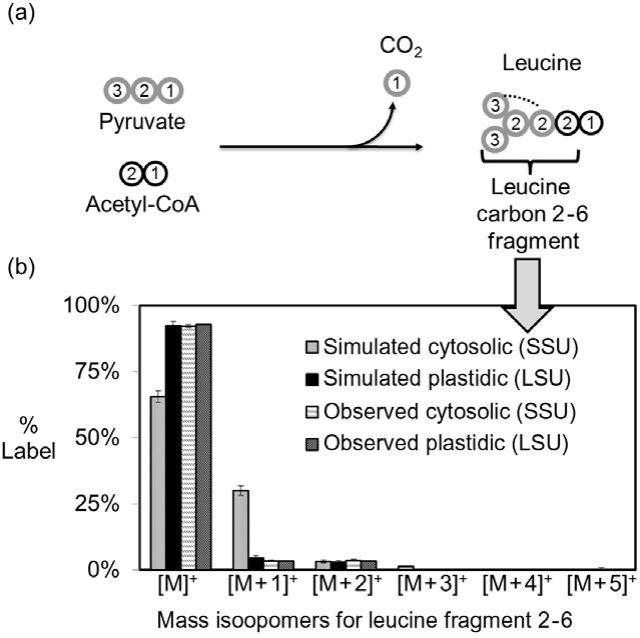
*In vivo* labelling confirms leucine biosynthesis in the plastid. The enrichment in leucine carbons 2–5 was determined from [U-^13^C]-glutamine labelling experiments (*n* = 3, SD). There were no statistical differences between the large subunit (LSU) and small subunit (SSU)-derived leucine, indicating that the cytosol and plastid pools are equivalent within the sensitivity of technique. (a) Schematic of leucine carbons derived from pyruvate and acetyl-CoA. (b) The expected enrichment for leucine biosynthesized in either plastid or cytosol was calculated from labelling in carbons derived from acetyl-CoA and pyruvate. The labelling in acetyl-CoA from fatty acid biosynthesis or elongation (see Fig. 4) was used to predict the ^13^C-label contribution to the first carbon of the leucine 2–5 fragment. Alanine carbons two and three (see Fig. 4) were used to represent ^13^C-labelling of pyruvate. The simulated labelling if carbons are derived from plastid pools matches the observed results. In contrast, labelling expected if synthesis occurs from cytosolic pools differs greatly from the observed results, particularly in the unlabelled ([M]^+^) and single labelled ([M+1]^+^) mass isotopomers. These results indicate that leucine is synthesized in the plastid and equilibrated across the plastid membrane to generate the cytosolic pool.

We further estimated these differences by simulating the labelling profile that is expected from precursors (Fig. 5). Using alanine as a readout for pyruvate and the average labelling from acetyl-CoA, we calculated the expected abundances of mass isotopomers for leucine made from cytosol and plastid precursors. The simulated values are compared with the measured values in the figure. The enriched cytosolic acetyl-CoA results in a simulated profile with more abundant [M+1]^+^ and reduced [M]^+^ compared with measured leucine. The simulation confirms that cytosolic acetyl-CoA was not used in leucine biosynthesis. Furthermore, the profile from plastid precursors is consistent with the measured enrichments for leucine from the LSU. These results provide *in vivo* confirmation that leucine biosynthesis occurs predominantly in the plastid, consistent with supporting evidence (Singh 1999). In addition, the data reinforce previous findings in *B. napus* embryos (Schwender *et al*. 2006) that acetyl-CoA pools are not in equilibrium between plastids and cytosol.

### Serine and glycine LSU and SSU pools are not in isotopic equilibrium

**Figure 6:**
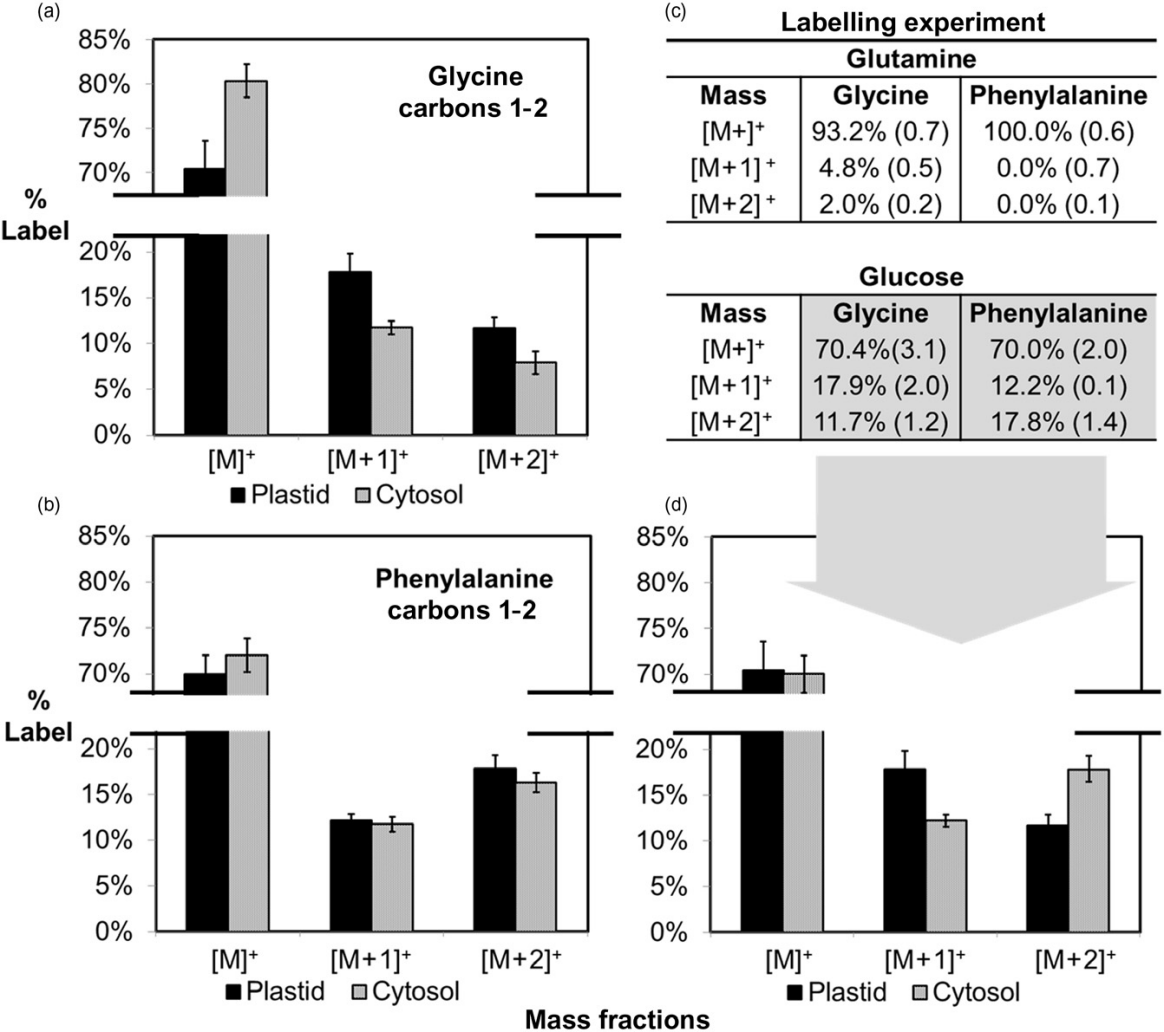
Glycine labelling differs from phenylalanine. Comparison of glycine and phenylalanine carbons from labelling experiments (*n* = 3, SD). Glycine and the first two carbons of phenylalanine are derived in part from three carbon intermediates including 3-phosphoglycerate and phospho*enol*pyruvate. (a) Glycine shows different mass enrichments between large subunits (LSUs) and small subunits (SSUs), (b) while phenylalanine is enriched similarly in both. (c) Glycine is significantly labelled by [U-^13^C]-glutamine whereas phenylalanine is unlabelled. (d) Glucose labelling reveals differences between glycine and phenylalanine that are derived from different metabolic processes.

Both serine and glycine are more highly ^13^C-enriched in LSU than in SSU (Fig. 2) and are labelled differently than phenylalanine. Phenylalanine is also made from triose (via phospho*enol*pyruvate) but does not display differences between LSU and SSU (Fig. 6), which probably reflects a single site of synthesis. The differences in average ^13^C enrichment for glycine and serine large and small subunits are similar to alanine (Fig. 2). However, unlike alanine, the maximally labelled [M+3]^+^ mass isotopomer in serine and [M+2]^+^ isotopomer in glycine are less abundant relative to other mass fractions (Fig. 6a and Supporting Information Tables S2–S4). The increased abundance of isotopomers with one or two ^13^C atoms indicates multiple bond-breaking events in metabolism and is indicative of pentose phosphate pathways as previously discussed. However, there are additional pathways for glycine and serine biosynthesis that may contribute to the isotopomer labelling patterns. For example, glycine labelling could be altered by reversible reactions through glycine oxidase, glycine decarboxylase, glycine dehydrogenase, glycine aminotransferase and/or serine hydroxyl-methyltransferase. The pathways result in partially labelled glycine but the extent of their subcellular activity in seeds is unknown. Of these possibilities, photorespiratory metabolism is unlikely because developing embryos of *B. napus* have concentrations of CO_2_ approaching 2000 times atmospheric levels (Goffman *et al*. 2005). Instead, the high CO_2_ levels may enhance the reversibility of glycine decarboxylase and other enzymes that utilize a CO_2_ substrate. Furthermore, one carbon groups are necessary for synthesis of other amino acids (i.e. histidine and methionine) and impact serine hydroxymethyltransferase and glycine dehydrogenase activities.

Interestingly, our results reveal that glycine also became labelled when cultures were provided with [U-^13^C]-glutamine (Fig. 6c). The absence of gluconeogenesis in filling oilseed embryos (Schwender *et al*. 2003; Allen *et al*. 2009b) implies that glycine label may come from threonine aldolase, utilizing threonine as a precursor. In comparison, the first two carbons of aromatic amino acids (i.e. that are derived from phospho*enol*pyruvate) remain unlabelled in the glutamine labelling experiment (Fig. 6c).

### LSU and SSU amino acids from aspartate and glutamate families are similarly labelled

**Figure 7:**
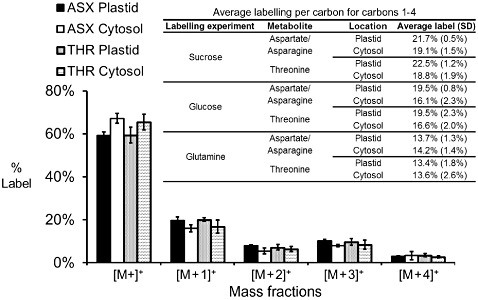
Labelling of four carbon products is similar across organelles. Comparison of aspartate/asparagine labelling with threonine from large subunits (LSUs) and small subunits (SSUs) (*n* = 3, SD). The labelling in four carbon amino acids is similar between subcellular compartments as indicated by label enrichment from [U-^13^C]-glucose. Labelling for other experiments is summarized in the inset table.

The ^13^C-enrichment of four and five carbon amino acids is presented in Fig. 7 and in Supporting Information Fig. S1, respectively. The aspartate family (aspartate, asparagine, methionine and threonine) is derived from oxaloacetate. The glutamate family (glutamate, glutamine, proline and arginine) is synthesized from glutamine supplied to the seed or from 2-oxoglutarate. These amino acids are tied to central metabolites of the TCA cycle and their biosynthesis is linked to enzymes with established subcellular locations such as phospho*enol*pyruvate carboxylase and succinate dehydrogenase. The results from ^13^C-glucose labelling reveal reduced differences in enrichment in isoleucine, aspartate/asparagine and threonine between LSU and SSU. Other labelling experiments presented in the figure reinforce these observations. The similar pool enrichments imply that either the amino acids or their precursors are isotopically equilibrated with regard to labelling across the plastid envelope.

## DISCUSSION

In this study, we investigated whether steady-state isotope labelling of proteins synthesized in different subcellular compartments would result in differential labelling of these proteins. We reasoned that if differences could be observed, these could provide a novel strategy to probe compartmentation of plant metabolism. Because Rubisco is a highly abundant protein with eight large and eight small subunits that are coordinately synthesized in the plastid and cytosol (Blair & Ellis 1973; Gray & Kekwick 1974; Baker *et al*. 1975), it was chosen as a test case for this study. Our results clearly indicate that some amino acids exhibit differences between the LSU and SSU in both the extent and the isotopomer patterns of ^13^C labelling. As described, these experiments have: (1) provided new information on the biosynthesis of alanine; (2) revealed that alanine, serine and glycine pools are not isotopically equilibrated between the cytosol and plastid; (3) confirmed *in vivo* that leucine synthesis is mostly confined to the plastid; and (4) provided evidence that amino acids from aspartate and glutamate families are nearly in isotopic equilibrium between the plastid and cytosol.

Other approaches have also provided *in vivo* information at the subcellular level. Compartment-specific labelling patterns for protein glycans have been established from hydroxyacetone and levulinic acid breakdown products using strong acid hydrolysis of protein (Sriram *et al*. 2007). Each of these labelled compounds reports specifically on cytosolic glycolysis. Starch that is synthesized in the plastid from ADP-glucose reflects the plastidic carbohydrate pool and has also been analysed by both GC-MS and nuclear magnetic resonance (NMR) (Allen *et al*. 2007). These methods have been used to resolve subcellular hexose phosphate pools. For example, Alonso *et al*. report a compartmentalized flux model of developing sunflower embryos based on the differences between starch and sucrose labelling (Alonso *et al*. 2007). Compared with starch, glycan and cell wall analysis that report on a limited number of precursor pools, amino acids provide more diverse information on primary metabolism.

When not overlooked, the successful engineering of metabolism at a compartmentalized level has provided major advantages. For example, the targeting of enzymes for the production of poly-hydroxybutyrate (PHB) to plastids of *Arabidopsis* resulted in a 100-fold increase in PHB accumulation compared with cytosolic targeting (Nawrath, Poirier & Somerville 1994). Nonetheless, such marked success stories are the exception, which may in part be because there are insufficient methods to assess metabolism at the subcellular level. This is one of the main challenges in producing validated flux models of plant seeds and other eukaryotic systems that include the compartmentation of metabolism (Kruger & Ratcliffe 2009; Allen *et al*. 2009a). Therefore, methods to assess the degree of compartmentation and biosynthetic location of metabolites, including amino acids, are important. In turn, these more accurate descriptions may help distinguish metabolic differences between wild type, mutants and transgenics that are of interest in basic biology and metabolic engineering. In combination with other compartmented analytes (such as the use of fatty acid labelling in this study) and flux analysis, the labelling in metabolic precursors in different compartments can be determined. Together, these approaches can be used to tackle other open questions about the compartmentation of amino acid metabolism in plants. For example, assessing the relative contributions of different compartments to the synthesis of the important amino acids cysteine (Hell & Wirtz 2011) and proline (Verslues & Sharma 2010) would be valuable and potentially accessible by modest modifications of the methods described here.

### Limitations of the study

In this study, enrichments in amino acids and their patterns of isotopomer labelling report on the isotopic equilibration of subcellular pools, and in some cases on possible difference in pathways for the biosynthesis of amino acids (or precursors) in different compartments. The technique establishes the degree of similarity of pools from different organelles and indicates when multiple pools may be involved. We note that if pools equilibrate fully across the plastid envelope, information on differences in metabolism in the plastid and cytosol is not available. Partial isotopic equilibration between compartments will also reduce labelling differences and therefore changes in enrichment are not necessarily proportional to the relative contribution of synthesis of an amino acid in different compartments. However, such exchange cannot create or increase labelling inequalities. Therefore, observed differences in labelling provide a clear indication of multiple origins of those amino acids. The comparison of these results to benchmark data on metabolite or enzyme localization can provide a guide for further experimentation.

The concentrations of enriched amino acids, labelled substrate selection and sensitivity of instrumentation are important. In this study, several amino acids could not be accurately compared between the LSU and SSU because of low concentrations or contaminations and losses during hydrolysis. Future more extensive analysis will be enabled by improving sensitivities of MS instruments.

### Outlook for and extension of methodology

Ribosomes and protein synthesis occur in mitochondria, cytosol and plastid in plants, and in mitochondria and cytosol of other eukaryotic organisms. Therefore, this method is extendable to essentially any eukaryote, where sufficient amounts of protein are available. In addition, the same concept can be applied to compare metabolism in specific cell types of multicellular organisms. For example, developing seeds are an organ with at least four cell types: seed coat, endosperm, embryo radical and embryo cotyledons. Mitochondrial multi-protein complexes with individual proteins produced in the cytosol or mitochondria would be present in all cell types and their examination would provide a strategy to examine whether subcellular metabolic fluxes are partitioned differently in these different cell types.

Even within a single tissue, cells are exposed to substantially different environments. For example, abaxial and adaxial cells of developing oilseed embryos differ by several-fold in ATP and oxygen levels, corresponding to light and oxygen availability (Vigeolas *et al*. 2003; Borisjuk *et al*. 2005; Rolletschek *et al*. 2005). Analysis of the Rubisco subunits from dissected sections of the seeds would provide further information on how these differences influence metabolism across a gradient of cells and within organelles.

## MATERIALS AND METHODS

### Chemicals

Methanol, hexane, isopropanol and acetonitrile, the labelled substrates: [U-^13^C]-glucose, [U-^13^C]-sucrose and [U-^13^C]-glutamine, and their unlabelled counterparts were all purchased from Sigma (Milwaukee, WI, USA). Hydrochloric acid was purchased from Mallinckrodt Baker (Phillipsburg, NJ, USA).

### Plant material and embryo culturing

Greenhouse cultivation and embryo culturing of canola embryos with labelled organic substrates have been previously described (Schwender *et al*. 2006) and were used with slight modifications. *B. napus* plants were grown at 22 °C in 3 gallon pots that contained a 3:1 mixture of soil : vermiculite in a greenhouse irradiated with sunlight and supplemental lights at ∼150 *µ*E to maintain at least 14 h of daylight conditions. Plants were watered three times daily and supplemented with Peters 20:20:20 nutrient solution. When embryos had developed on the plant to a torpedo stage, siliques were removed and surface sterilized in a 5% hypochlorite solution and rinsed with water. Seeds were removed from siliques and the seed coat was removed aseptically and embryos were transferred to Petri dishes for culture duration. The culture medium composition has been previously described (Schwender *et al*. 2003) and is composed of sucrose (80 mm), glucose (40 mm), glutamine (35 mm) and alanine (10 mm). For each labelling experiment, labelled substrates were substituted for 100% of their unlabelled counterpart, except for sucrose in which labelled sucrose replaced 50% of total sucrose. A complete list of macro- and micronutrients supplemented with cultures is given elsewhere (Schwender *et al*. 2003) and Gamborg's vitamins (Sigma) were also provided. Approximately 36 embryos were cultured in six Petri dishes for 14 d. Harvested embryos were rinsed in 1 L of water and either processed immediately or frozen in liquid nitrogen and stored at −80 °C.

### Protein extraction, purification and hydrolysis

Embryos were placed in 1.5 mL tubes and extracted on a Retsch MM 301 Bead Beater using one steel ball and 0.6 mL of protein extraction buffer containing: 10 mm Tris-HCl, 150 mm NaCl, pH 7.4 and 60 *µ*L of protease inhibitor stock (1 mini EDTA-free tablet per 500 *µ*L water, Roche Indianapolis, IN, USA, #13821400). After 60 min at 30 Hz, the tubes were centrifuged at ∼13 200 *g* for 12 min at 4 °C and supernatant was directly loaded on a Seppro IgY-Rusbico Spin affinity purification column (GenWay Biotech Inc 28–288-23153-SC, San Diego, CA or Sigma). The extract and affinity beads were agitated overnight at 4 °C on an orbital shaker at low speed. Then the columns were washed of unbound proteins using extraction buffer and the intact Rubisco was recovered using a stripping buffer containing: 100 mm Glycine-HCl pH 2.5 and 10% v/v protease inhibitor stock. Affinity-purified Rubisco was subsequently processed through SDS-PAGE using 10.5–14% Tris-HCl Precast criterion gels (345-9949 Bio-Rad, Hercules, CA, USA) using a Bio-Rad Power Pac Universal Power Supply. Because the fractions of Rubisco from the column were dilute (∼0.3 *µ*g *µ*L^−1^), protein was first concentrated on the gel by loading 45 *µ*L, running the gel for 40–60 s and reloading with more extract to focus a large amount of Rubisco within the stacking gel.

Using molecular weight markers and Rubisco harvested from leaf as standards, the large and small subunits of Rubisco were confirmed with Coomassie blue staining. Gels were electroblotted (Bio-Rad Criterion Cell) to membranes (Bio-Rad Immun-blot PVDF Membrane) overnight for ∼18 h at 15 V followed by 1 h at 100 V at 4 °C using a transfer buffer composed of 10 mm CAPS (pH 11), 10% methanol (v : v). Membranes were very briefly developed using 5% Coomassie blue stain contained in a 50% methanol, 10% acetic acid solution (Sigma). Next, bands were immediately and thoroughly destained by incubating in alternate solutions of water and methanol. Protein bands were excised and further washed. Protein amino acids were hydrolyzed from gel slices at 110 °C for 28 h in the presence of 6N HCl in an oxygen-deficient environment, dried by evaporation with nitrogen at 60 °C and stored at −20 °C until subsequent derivatization. Cation exchange was tested but did not further improve amino acid purity, reduced yields and was deemed unnecessary for processing by GC-MS.

### Butyl amide reactions

Butyl amides were generated as has been previously described (Allen *et al*. 2007). Oil was extracted from *Brassica* embryos using a 2:1 (v : v) mixture of hexane : isopropanol. The extracted oil was reacted with 2 mL n-butylamine (Sigma) in the presence of 3 mL hexane. Reactions proceeded for 48 h at 75 °C and were quenched by the addition of concentrated HCl that resulted in phase partitioning of unreacted butylamine from hexane and butylamides. The non-polar hexane phase was concentrated by evaporation under nitrogen before GC-MS analysis. Specific details to GC-MS settings can be found elsewhere (Allen *et al*. 2007).

### GC-MS of amino acids

For ^13^C labelling, protein amino acids were converted to their tert-butyl-dimethylsilyl derivatives using MTBSTFA (Sigma) and analysed with GC-MS (Das Neves & Vasconcelos 1987; Dauner, Bailey & Sauer 2001). A Thermo – Trace Ultra gas chromatogram linked to a DSQII mass spectrometer and a TriPlus autosampler was used for analysis. A DB5 (30 m × 0.2 mm × 0.33 *µ*m) silica column was used for separations. The column utilized a helium carrier gas and split injections of 10:1 (injection temperature 250 °C, detector temperature 280 °C). The temperature profile consisted of a starting temperature of 40 °C for 1 min followed by a 50 °C per min ramp to 100 °C, a hold time of 1 min, then 5 °C per min to a temperature of 200 °C, followed by 10 °C per min to a temperature of 300 °C. The EI (70 eV) MS was tuned before runs using the m/z 69, 219, and 502 ions of perflurotributylamine as a calibrant. Total ion chromatograms were used to scan the weights from 50 to 500 m/z and establish retention times that were then used in coordination with selected ion monitoring to quantitate the relative labelling. Ions of interest and correction for natural abundance have been described elsewhere (Antoniewicz, Kelleher & Stephanopoulos 2007; Allen *et al*. 2009b).

## SUPPORTING INFORMATION

**Figure S1.** Labelling in glutamate-derivedcompounds is equivalent across organelles. Glutamate-derivedproducts proline and glutamine/glutamate are equivalently labelledfor multiple labelling experiments (*n* = 3, SD). Arginine enrichments were qualitatively similar but not reported because of complex fragmentation patterns described elsewhere (Allen & Ratcliffe 2009).

**Table S1.** Subcellular biosynthetic origin ofamino acid pathway enzymes. Information on the subcellularlocalization in *Arabidopsis* of the indicated amino acid biosynthesis enzymes was derived from a number of literature sources and websites, including the examples given. Multiple locations reported for a number of enzymes indicate that the biosynthetic locations of amino acids are either not conclusive or suggest that amino acids can be synthesized in multiple subcellular compartments.

**Table S2.** Average carbon labelling for LSU and SSU. The average labelling per carbon was calculated as described in Fig. 2 of the main text and the differences were compared between the LSU and SSU that represent plastidic and cytosolic pools, respectively.

**Table S3.** Isotope fractional labelling in (a)plastidic and (b) cytosolic amino acids after provision of[U-^13^C]-glucose to filling *B. napus* embryos.

**Table S4.** Isotope fractional labelling in (a)plastidic and (b) cytosolic amino acids after provision of[U-^13^C]-sucrose to filling *B. napus* embryos.

**Table S5.** Isotope fractional labelling in (a)plastidic and (b) cytosolic amino acids after provision of[U-^13^C]-glutamine to filling *B. napus* embryos.

**Table S6.** Isotope fractional labelling in (a)plastidic and (b) cytosolic amino acids after provision of[U-^13^C]-alanine to filling *B. napus* embryos.

**Appendix S1.** Supplement references.
