# Supplementary material for: Isotope labelling of Rubisco subunits provides in vivo information on subcellular biosynthesis and exchange of amino acids between compartments
Source: Plant Cell Environ. 2012 Jul;35(7):1232–44. doi: 10.1111/j.1365-3040.2012.02485.x (PMC3556518; doi:10.1111/j.1365-3040.2012.02485.x)
Supplement: Supplementary file 1 [file pce0035-1232-SD1.doc]

**Isotope labeling of RuBisCO subunits provides in vivo information on subcellular biosynthesis and exchange of amino acids between compartments**

**Doug K. Allen*, Russell W. LaClair, John B. Ohlrogge, Yair Shachar-Hill**

**SUPPLEMENT**

**Supplement Table 1.** Subcellular biosynthetic origin of amino acid pathway enzymes. Information on the subcellular localization in Arabidopsis of the indicated amino acid biosynthesis enzymes was derived from a number of literature sources and websites, including the examples given. Multiple locations reported for a number of enzymes indicate the biosynthetic locations of amino acids are either not conclusive or suggests that amino acids can be synthesized in multiple subcellular compartments.

| **Amino Acid** | **Biosynthetic Enzyme** | **Locus ID** | **Subcellular Detail** | **Example References** |
| --- | --- | --- | --- | --- |
| **Alanine** | Alanine aminotransferase | AT1G17290/  AT1G72330 | Mitochondrion, chloroplast | Lee et al. 2008; Ito et al. 2006;  Kleffmann et al. 2004 |
| **Arginine** | Arginosuccinate lyase/synthase | AT5G10920/AT4G24830 | Chloroplast | Zybailov et al. 2008; Peltier et al. 2006; Kleffmann et al. 2004 |
| **Asparagine** | Asparagine synthase | *Pea ref. | Cytosol | Ireland & Joy, 1983 |
| **Aspartate** | Aspartate aminotransferase | AT2G30970/AT4G31990/ AT5G19550 | Mitochondrion, chloroplast, cytosol | Lee et al. 2008; Kleffmann et al. 2004; Marmagne et al. 2007; Ito et al. 2011 |
| **Cysteine** | Serine O- acetyltransferase/  acetylserine-lyase | AT1G55920AT2G43750, AT3G59760 | Cytosol, Chloroplast, nucleus, mitochondrion | Kleffmann et al. 2004; Bae et al. 2003; Froehlich et al. 2003; Peltier et al. 2006; Zybailov et al. 2008; Ito et al. 2006 |
| **Glutamate** | Glutamate synthase | AT5G53460 | Chloroplast | Kleffmann et al. 2004; Zybailov et al. 2008 |
| **Glutamine** | Glutamine synthetase | AT1G48470, AT5G35630 | Chloroplast, cytosol | Zybailov et al. 2008; Froehlich et al. 2003; Peltier et al. 2006; Giavalisco et al. 2005 |
| **Glycine** | Serine/glycine hydroxyl-methyl-transferase | AT4G32520, AT4G37930 | Chloroplast, mitochondrion, nucleus, cytosol | Zybailov et al. 2008; Kleffmann et al. 2004; Lee et al. 2008; Bae et al. 2003;  Giavalisco et al. 2005 |
| **Histidine** | phosphoribosyl transferase/ phosphoribosyl pyrophospho-hydrolase | AT1G09795,  AT1G31860 | Chloroplast | Zybailov et al. 2008 |
| **Isoleucine, Leucine, Valine** | Acetolactate synthase, ketol-acid reductoisomerase | AT3G48560, AT3G58610 | Chloroplast | Zybailov et al. 2008; Froehlich et al. 2003; Peltier et al. 2006; Kleffmann et al. 2004 |
| **Lysine** | Dihydrodipicolinate synthase, Diaminopimelate decarboxylase | AT2G45440  AT3G14390, AT5G11880 | Chloroplast | Zybailov et al. 2008 |
| **Methionine** | Methionine synthase | AT5G20980 | Chloroplast, cytosol | Zybailov et al. 2008; Ravanel et al. 2004; de la Fuente van Bentem et al. 2008 |
| **Phenylalanine, Tyrosine** | Chorismate mutase, 3-dehydroquinate synthase | AT5G10870, AT5G66120 | Chloroplast, cytosol | Zybailov et al. 2008; Eberhard et al 1996 |
| **Proline** | Pyrroline-5-carboxylate reductase/ delta1-pyrroline-5-carboxylate synthase | AT5G14800, AT2G39800 | Cytosol, chloroplast | TIGR annotation; Szekely et al. 2008 |
| **Serine** | Phosphoserine phosphatase, 3-phosphoglycerate dehydrogenase | AT1G18640  AT1G17745 | Chloroplast, cytosol, nucleus | Ho et al. 1999; Zybailov et al. 2008; de la Fuente van Bentem et al. 2008 |
| **Threonine** | Homoserine dehydrogenase  Threonine synthase  Homoserine kinase | AT1G31230,  AT1G72810, AT4G29840, AT2G17265 | Chloroplast | TAIR; Zybailov et al. 2008;  Rutschow et al 2008 |
| **Tryptophan** | Anthranilate synthase  Tryptophan synthase | AT1G24807, AT2G29690, AT3G54640 | Chloroplast | Zybailov et al. 2008, Peltier et al. 2006 |

**Supplement Table 2.** Average carbon labeling for LSU and SSU. The average labeling per carbon was calculated as described in Table 2 of the main text and the differences were compared between the LSU and SSU that represent plastidic and cytosolic pools respectively.

|  | | | | |  |  |  |  |
| --- | --- | --- | --- | --- | --- | --- | --- | --- |
| **Labeling Experiment** | **[U-^13^C]-sucrose** | | **[U-^13^C]-glucose** | | **[U-^13^C]-glutamine** | | **[U-^13^C]-alanine** | |
| **Amino Acid** | **LSU** | **SSU** | **LSU** | **SSU** | **LSU** | **SSU** | **LSU** | **SSU** |
| Alanine | 24% | 17% | 21% | 16% | 1% | 2% | 15% | 15% |
| Glycine | 22% | 15% | 20% | 14% | 4% | 5% | 1% | 1% |
| Leucine | 30% | 28% | 24% | 22% | 2% | 3% | 4% | 4% |
| Isoleucine | 26% | 24% | 22% | 21% | 10% | 11% | 2% | 2% |
| Proline | 9% | 8% | 10% | 9% | 61% | 57% | 3% | 3% |
| Glu/Gln | 9% | 7% | 9% | 8% | 57% | 61% | 2% | 2% |
| Serine | 20% | 12% | 20% | 14% | 2% | 3% | 0% | 0% |
| Threonine | 22% | 19% | 19% | 17% | 13% | 14% | 2% | 2% |
| Phenylalanine | 32% | 30% | 24% | 23% | 0% | 0% | 0% | 0% |
| Asp/Asn | 21% | 19% | 20% | 17% | 14% | 14% | 1% | 2% |

**Supplement Table 3.** Isotope fractional labeling in (a) plastidic and (b) cytosolic amino acids after provision of [U-^13^C]-glucose to filling *B. napus* embryos.


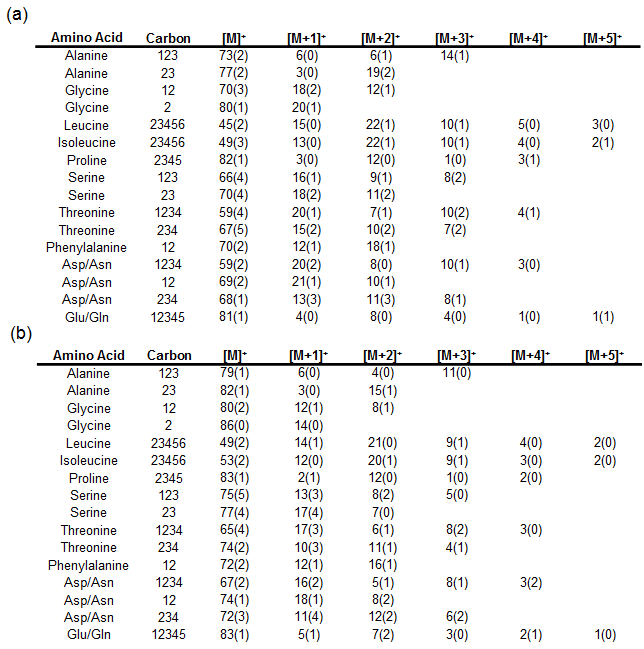


**Supplement Table 4.** Isotope fractional labeling in **a)** plastidic and **b)** cytosolic amino acids after provision of [U-^13^C]-sucrose to filling *B. napus* embryos.


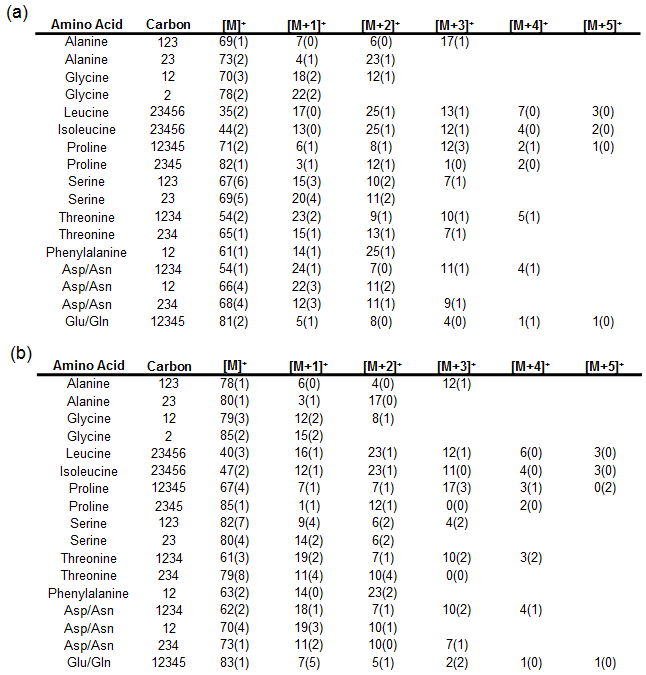


**Supplement Table 5.** Isotope fractional labeling in **a)** plastidic and **b)** cytosolic amino acids after provision of [U-^13^C]-glutamine to filling *B. napus* embryos.


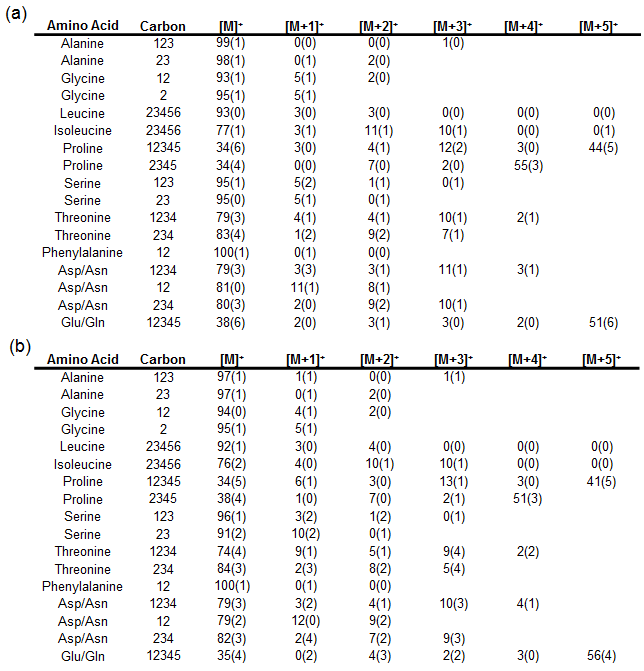


**Supplement Table 6.** Isotope fractional labeling in **a)** plastidic and **b)** cytosolic amino acids after provision of [U-^13^C]-alanine to filling *B. napus* embryos.


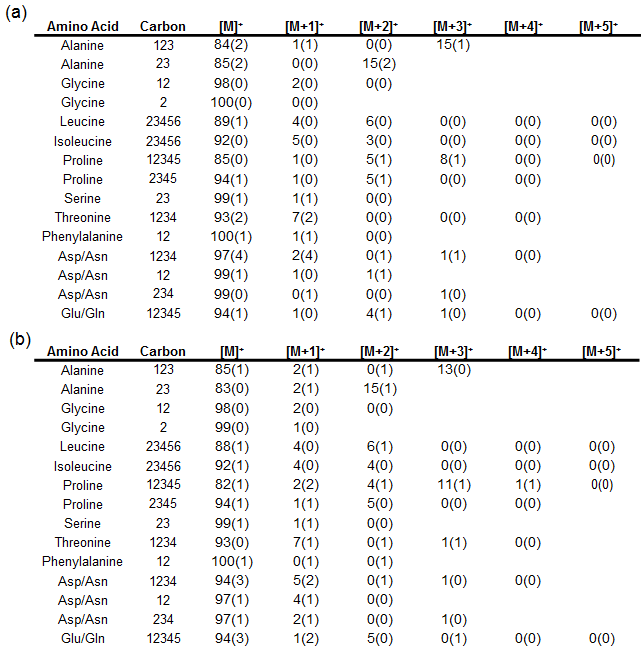


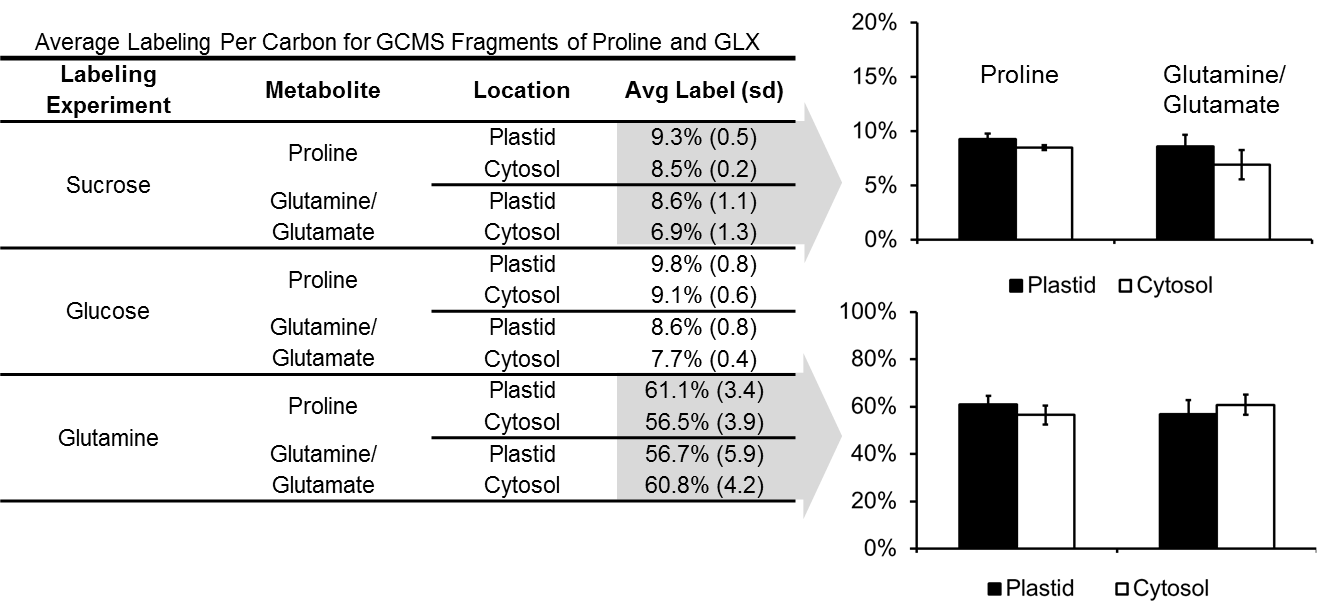


**Supplement Figure 1.** Labeling in glutamate derived compounds is equivalent across organelles. Glutamate-derived products proline and glutamine/glutamate are equivalently labeled for multiple labeling experiments (n=3, SD). Arginine enrichments were qualitatively similar but not reported because of complex fragmentation patterns described elsewhere .

**Supplement** **References:**

1. Lee CP, Eubel H, O'Toole N, Millar AH (2008) Heterogeneity of the mitochondrial proteome for photosynthetic and non-photosynthetic Arabidopsis metabolism. Molecular and Cellular Proteomics 7: 1297-1316.

2. Ito J, Heazlewood JL, Millar AH (2006) Analysis of the soluble ATP-binding proteome of plant mitochondria identifies new proteins and nucleotide triphosphate interactions within the matrix. Journal of Proteome Research 5: 3459-3469.

3. Kleffmann T, Russenberger D, Von Zychlinski A, Christopher W, Sjölander K, et al. (2004) The Arabidopsis thaliana chloroplast proteome reveals pathway abundance and novel protein functions. Current Biology 14: 354-362.

4. Zybailov B, Rutschow H, Friso G, Rudella A, Emanuelsson O, et al. (2008) Sorting signals, N-terminal modifications and abundance of the chloroplast proteome. PLoS ONE 3.

5. Peltier JB, Yang C, Qi S, Zabrouskov V, Giacomelli L, et al. (2006) The oligomeric stromal proteome of Arabidopsis thaliana chloroplasts. Molecular and Cellular Proteomics 5: 114-133.

6. Ireland RJ, Joy KW (1983) Subcellular Localization of Asparaginase and Asparagine Aminotransferase in Pisum sativum Leaves. Plant Physiology 72: 1127-1129.

7. Marmagne A, Ferro M, Meinnel T, Bruley C, Kuhn L, et al. (2007) A high content in lipid-modified peripheral proteins and integral receptor kinases features in the Arabidopsis plasma membrane proteome. Molecular and Cellular Proteomics 6: 1980-1996.

8. Ito J, Batth TS, Petzold CJ, Redding-Johanson AM, Mukhopadhyay A, et al. (2011) Analysis of the Arabidopsis cytosolic proteome highlights subcellular partitioning of central plant metabolism. Journal of Proteome Research 10: 1571-1582.

9. Bae MS, Cho EJ, Choi EY, Park OK (2003) Analysis of the Arabidopsis nuclear proteome and its response to cold stress. Plant Journal 36: 652-663.

10. Froehlich JE, Wilkerson CG, Ray WK, McAndrew RS, Osteryoung KW, et al. (2003) Proteomic study of the Arabidopsis thaliana chloroplastic envelope membrane utilizing alternatives to traditional two-dimensional electrophoresis. Journal of Proteome Research 2: 413-425.

11. Giavalisco P, Wilson D, Kreitler T, Lehrach H, Klose J, et al. (2005) High heterogeneity within the ribosomal proteins of the Arabidopsis thaliana 80S ribosome. Plant Molecular Biology 57: 577-591.

12. Ravanel S, Block MA, Rippert P, Jabrin S, Curien G, et al. (2004) Methionine metabolism in plants: Chloroplasts are autonomous for de novo methionine synthesis and can import S-adenosylmethionine from the cytosol. Journal of Biological Chemistry 279: 22548-22557.

13. De La Fuente Van Bentem S, Anrather D, Dohnal I, Roitinger E, Csaszar E, et al. (2008) Site-specific phosphorylation profiling of arabidopsis proteins by mass spectrometry and peptide chip analysis. Journal of Proteome Research 7: 2458-2470.

14. Eberhard J, Ehrler TT, Epple P, Felix G, Raesecke HR, et al. (1996) Cytosolic and plastidic chorismate mutase isozymes from Arabidopsis thaliana: Molecular characterization and enzymatic properties. Plant Journal 10: 815-821.

15. Székely G, Ábrahám E, Cséplo Á, Rigó G, Zsigmond L, et al. (2008) Duplicated P5CS genes of Arabidopsis play distinct roles in stress regulation and developmental control of proline biosynthesis. Plant Journal 53: 11-28.

16. Ho CL, Noji M, Saito K (1999) Plastidic pathway of serine biosynthesis: Molecular cloning and expression of 3-phosphoserine phosphatase from Arabidopsis thaliana. Journal of Biological Chemistry 274: 11007-11012.

17. Rutschow H, Ytterberg AJ, Friso G, Nilsson R, Van Wijk KJ (2008) Quantitative proteomics of a chloroplast SRP54 sorting mutant and its genetic interactions with CLPC1 in arabidopsis. Plant Physiology 148: 156-175.

18. Allen D.K. & Ratcliffe R.G. (2009) Quantification of Isotope Label. In: *Plant Metabolic Networks* (ed J. Schwender), pp. 105-149. Springer, New York.
